# Gambogenic Acid Suppresses Malignant Progression of Non-Small Cell Lung Cancer via GCH1-Mediated Ferroptosis

**DOI:** 10.3390/ph18030374

**Published:** 2025-03-06

**Authors:** Menghan Wang, Jiao Liu, Wenxi Yu, Jiancang Shao, Yang Bao, Mingming Jin, Qingqing Huang, Gang Huang

**Affiliations:** 1 Graduate School of Shanghai University of Traditional Chinese Medicine, Shanghai 201203, China; wangmenghan430@163.com (M.W.);; 2 Shanghai Key Laboratory of Molecular Imaging, Shanghai University of Medicine and Health Sciences, Shanghai 201318, China

**Keywords:** gambogenic acid, non-small cell lung cancer, ferroptosis, GCH1

## Abstract

**Introduction:** Non-small cell lung cancer (NSCLC) is a lethal type of lung cancer (LC) with a 5-year survival rate of 19%. Because drug resistance typically develops following chemotherapy, radiotherapy, and immunotherapy, a novel NSCLC therapeutic strategy is urgently demanded. Gambogenic acid (GNA), a major bioactive ingredient isolated from gamboge, has multipotent antitumor effects, although activity against NSCLC is unknown. **Methods:** CCK8, ethynyl deoxyuridine (EdU), the plate colony formation assay, and the transwell and wound healing (WH) assay were used to study the effect of GNA on the proliferation and migration ability of NSCLC. Flow cytometry was used to detect apoptosis and the cell cycle. Proteomic analysis and LiP-SMap were used to detect the downstream target of GNA. Ferroptosis inhibitor ferrostatin-1 was used to detect the effect of GNA on NSCLC ferroptosis. Overexpressing GCH1 was used for a rescue experiment. Subcutaneous tumor and pulmonary metastasis in a mouse model were used to study the effect of GNA on NSCLC growth and metastasis. **Results:** The results of the present study showed that GNA inhibited the proliferation and migration of NSCLC cells in a dose- and time-dependent manner, which arrested the cell cycle in the G0/G1 phase. In vivo data revealed that GNA inhibited tumor growth and lung metastasis. Proteomic analysis found that GNA significantly inhibited the expression of GTP cyclohydrolase 1 (GCH1). LiP-SMap analysis showed that GNA interacted with ILE248 and ARG249 of GCH1. GCH1 overexpression had a similar role to the ferroptosis inhibitor ferrostatin-1 and restored cell proliferation and migration after GNA treatment. Also, GNA promoted reactive oxygen species (ROS) accumulation, which reduced mitochondrial membrane potential. GCH1 overexpression or ferrostatin-1 treatment reversed GNA regulation of ROS accumulation and mitochondrial membrane potential inhibition. **Conclusions:** Taken together, these findings confirmed that GNA suppressed the malignant progression of NSCLC by inducing GCH1-mediated ferroptosis.

## 1. Introduction

Non-small cell lung cancer (NSCLC) is the most common and lethal type of lung cancer (LC) [1]. Significant advances in screening and diagnosis have revolutionized treatment of NSCLC over the past decade [2,3]. Nevertheless, the 5-year survival rate regarding NSCLC is 19%. Because drug resistance typically develops following chemotherapy, radiotherapy, and immunotherapy, novel therapeutic strategies for NSCLC are urgently needed [4].

Ferroptosis is an iron-dependent form of programmed cell apoptosis, which is broadly regarded as the main reason leading to tumorigenesis and cancer progression [5,6,7,8]. Prior studies have identified several shared initiators and pathways of ferroptosis and epithelial–mesenchymal transition, suggesting potential as prognostic indicators among different cancers [7,9,10,11].

Ethanolic extract of gamboge is a brittle gum resin gained from different Southeast Asian trees in the genus *Garcinia* that has broad-spectrum anticancer activities, and the main active ingredient, gambogenic acid (GNA), has been formulated into injectable formulations for Phase II clinical trials in China [12] to induce apoptosis and inhibit proliferation in different cancer types, including melanoma, colorectal cancer, osteosarcoma, and glioma [13,14,15,16]. Previous investigations confirmed that GNA is a potential anticancer therapeutic agent, which suppresses proliferation and ferroptosis by targeting the AMPKα/SLC7A11/GPX4 and miR-1291/FOXA2 axes [17].

Nevertheless, the regulatory mechanisms associated with GNA in NSCLC remain unknown. The current study aimed to reveal the mechanisms of GNA-induced death of A549 and HCC1833 cells. Data revealed that GNA effectively suppressed tumor growth and metastasis. More importantly, GNA influenced redox levels by reducing GCH1 expression, which further promoted ferroptosis of tumor cells, as also confirmed by rescue experiments. The data shed new light on the mechanisms regarding GNA-mediated death of NSCLC cells.

## 2. Results

### 2.1. GNA Inhibited Proliferation and Migration of NSCLC Cells

**Figure 1:**
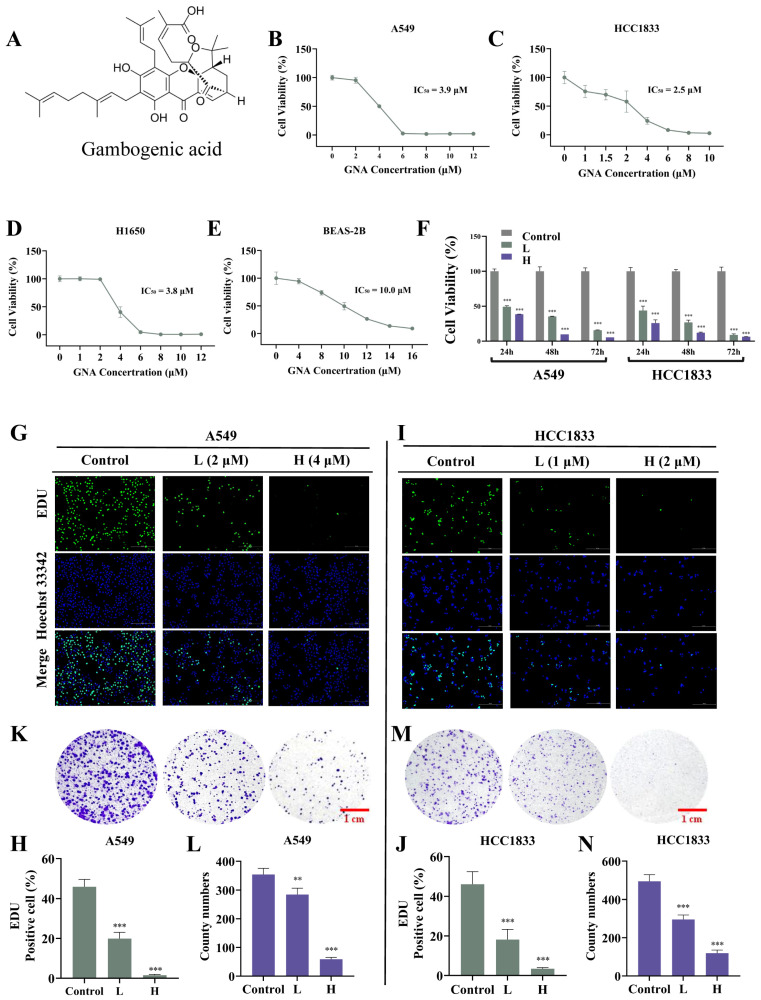
GNA treatment inhibits proliferation ability of NSCLC cells. (**A**–**E**) CCK-8 results for proliferation ability of A549, HCC1833, H1650, and BEAS-2B cells after treatment with different GNA doses for 24 h. (**F**) CCK-8 results for proliferation ability of A549 and HCC1833 cells after treatment with IC50 dose of GNA lasting for 24, 48, and 72 h. Data are expressed as mean ± SD. (**G**–**J**) EdU results for proliferation ability of A549 and HCC1833 cells (20×). Data are expressed as mean ± SD. (**K**–**N**) Colony formation results for proliferation ability of A549 and HCC1833 cells. Data are expressed as mean ± SD. ** *p* < 0.01, *** *p* < 0.001 vs. control.

**Figure 2:**
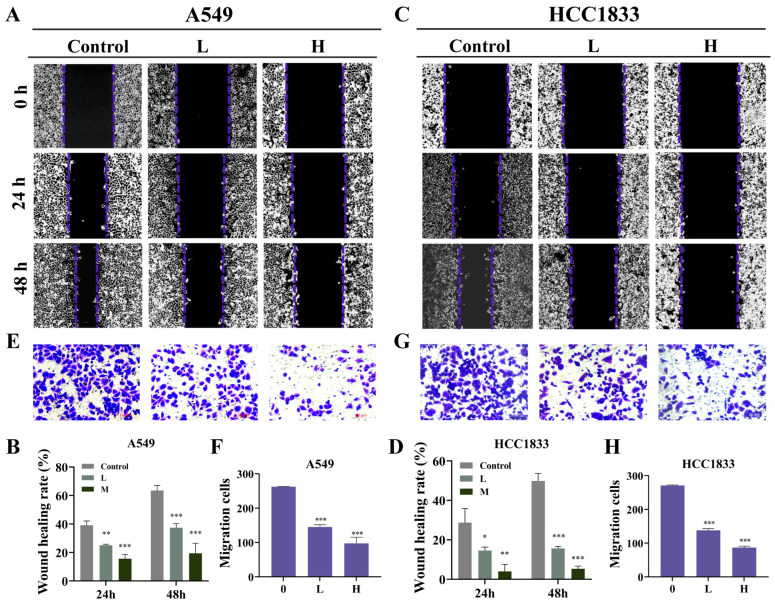
GNA treatment inhibits migration ability of NSCLC cells. (**A**–**D**) WH results for migration ability of A549 and HCC1833 cells after treatment with different GNA doses for 0, 24, and 48 h (10×). (**E**–**H**) Transwell assay results for migration ability of A549 and HCC1833 cells (20×). Data are expressed as mean ± SD. * *p* < 0.05, ** *p* < 0.01, *** *p* < 0.001 vs. control.

The chemical structure of GNA is shown in Figure 1A. Several investigations have confirmed the antitumor effects of GNA [18]. In the current research, CCK-8 assay data verified that GNA suppressed proliferation regarding A549, HCC1833, H1650, and pulmonary epithelial BEAS-2B cells, with half-maximal inhibitory concentrations of 3.9, 2.5, 3.8, and 10 μM, respectively (Figure 1B–E), suggesting that non-small cell lung cancer cells were more sensitive to GNA than pulmonary epithelial BEAS-2B cells in a certain concentration range. A549 and HCC1833 cells were selected for the following study, which showed that GNA (A549: L, 2 μM; H, 4 μM. HCC1833: L, 1 μM; H, 2 μM) treatment significantly inhibited the proliferation ability of NSCLC cells in a time-dependent manner (Figure 1F). EdU assay data (Figure 1G–J) and colony formation assays (Figure 1K–N) verified that GNA significantly suppressed HCC1833 and A549 cell proliferation in a time- and dose-dependent manner. The WH assay (Figure 2A–D) and transwell assay (Figure 2E–H) found that GNA inhibited the migration of both A549 and HCC1833 cells.

### 2.2. GNA Can Regulate the Cell Cycle and Apoptosis

**Figure 3:**
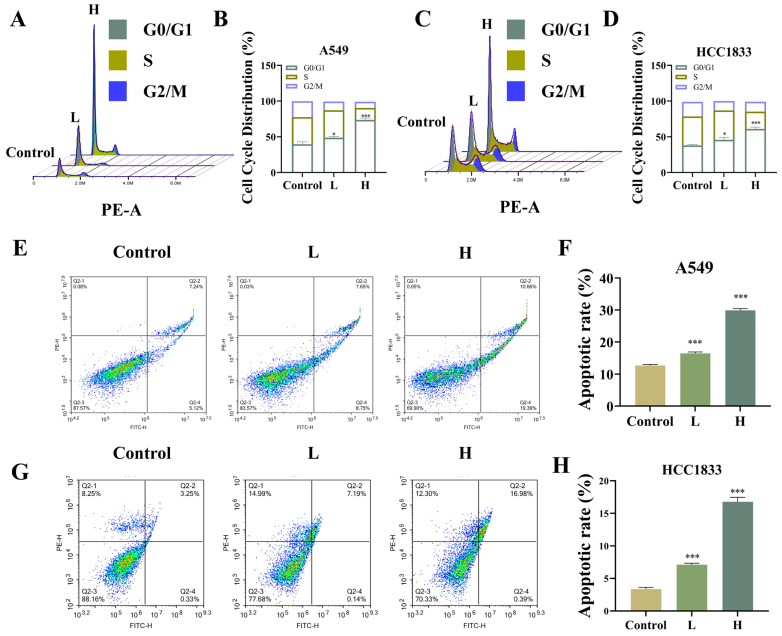
Effect of GNA on cell cycle and apoptosis regulation. (**A**–**D**) Cell cycle and (**E**–**H**) apoptosis were detected using flow cytometry in A549 and HCC1833 cells. Data are expressed as mean ± SD. * *p* < 0.05, *** *p* < 0.001 vs. control.

Cell cycle assay data showcased that GNA arrested cycles of A549 and HCC1833 cells in G0/G1 phase (Figure 3A–D), while the apoptosis assay using flow cytometry demonstrated that GNA promoted A549 and HCC1833 cell apoptosis (Figure 3E–H).

### 2.3. GNA Suppressed Tumor Growth and Lung Metastasis

**Figure 4:**
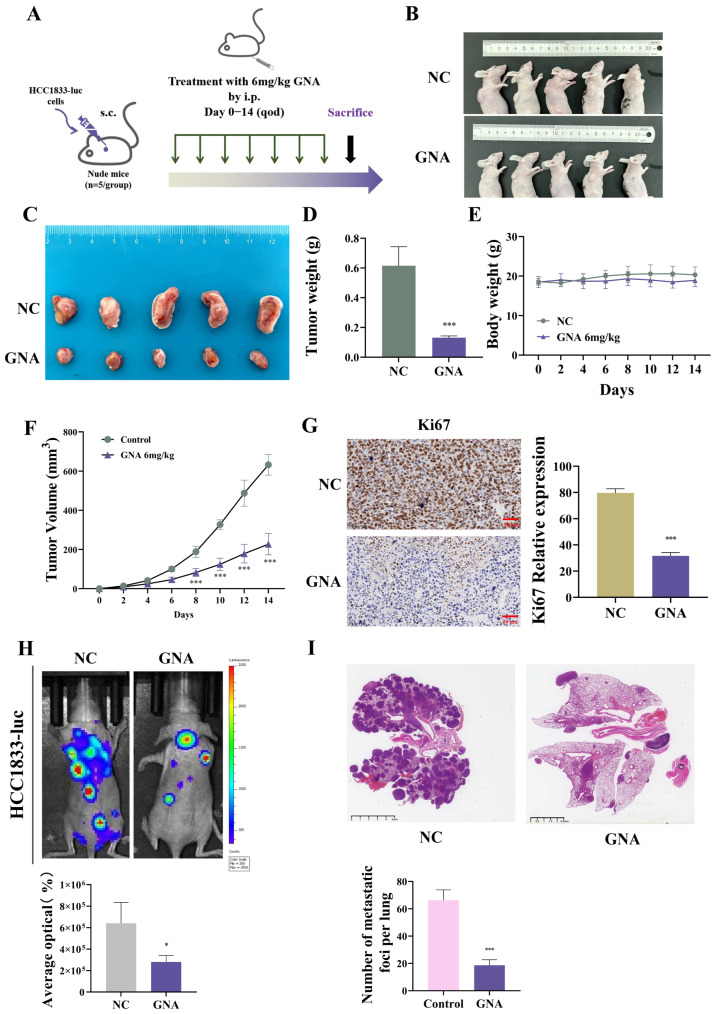
Effect of GNA on tumor growth and lung metastasis in vivo. (**A**) Schematic diagram illustrating nude mouse xenografts. (**B**,**C**) Representative HCC1833 tumor formation images in nude mouse xenografts. (**D**) Tumor weight was determined 14 days after injection. (**E**) Mouse body weight was determined every two days for 14 days. (**F**) Tumor volumes were recorded every two days. (**G**) Immunohistochemical analysis showing percentage of Ki67-positive cells. (**H**) Fluorescence density was determined using in vivo imaging. (**I**) HE staining results for tumor lesion numbers. Data are expressed as mean ± SD. * *p* < 0.05, *** *p* < 0.001 vs. control.

HCC1833 cells were used for tumor growth and lung metastasis experiments (Figure 4A). The results showed that GNA inhibited tumor growth (Figure 4B,C) and decreased tumor weight (Figure 4D) but did not influence the mice’s body weight (Figure 4E). Furthermore, GNA inhibited tumor growth by decreasing the tumor volume (Figure 4F). Immunohistochemical analysis revealed that GNA suppressed Ki67 expression (Figure 4G).

Live imaging of HCC-1833-luc cells showed that GNA reduced pulmonary metastases and metastatic foci counts in the lung (Figure 4H,I), suggesting that GNA suppressed proliferation and invasion of NSCLC cells.

### 2.4. GNA Inhibited GCH1 Expression

**Figure 5:**
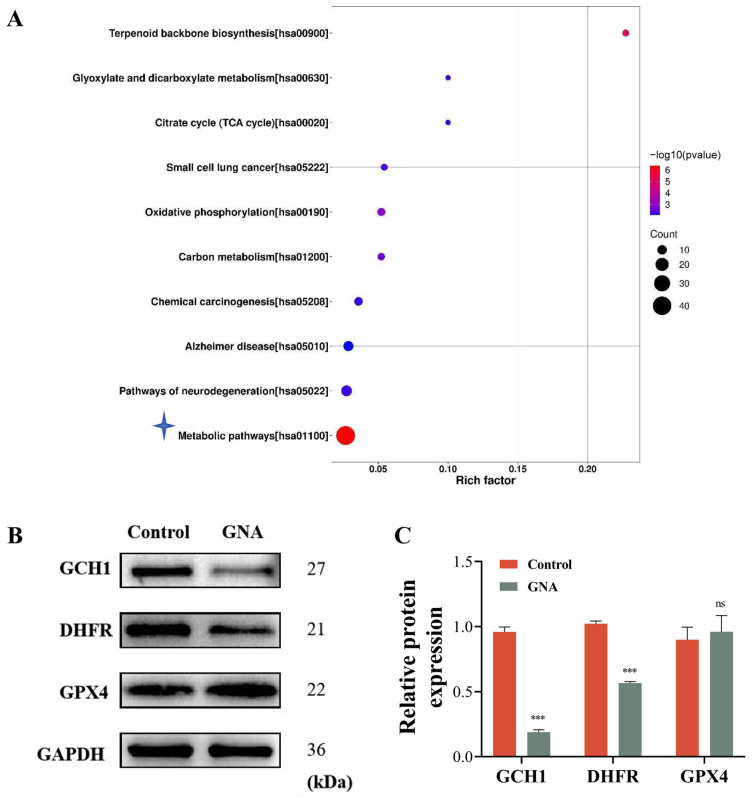
GNA treatment inhibits GCH1 expression. (**A**) KEGG analysis showing proteomic results after treatment with or without GNA in HCC1833 cells. (**B**,**C**) Western blot results for GCH1, DHFR, and GPX4 expression in HCC1833 with or without GNA treatment. Data are expressed as mean ± SD. ns: no significance, *** *p* < 0.001 vs. NC.

Proteomic analyses were conducted using HCC1833 cells treated with or without GNA to determine the regulatory mechanism. The data verified that GNA treatment led to metabolic dysfunction (Figure 5A). Among the more differentially expressed proteins, GCH1, a member of the ferroptosis antioxidant defense system [19], is associated with BH4 metabolism [20]. Therefore, we hypothesized that GNA could induce ferroptosis of tumor cells by inhibiting GCH1 expression and disrupting tumor REDOX homeostasis. WB data illustrated that GNA reduced GCH1 and DHFR expression levels, which are both key enzymes of BH4 biosynthesis (Figure 5B,C).

### 2.5. GCH1 Is the Downstream Target of GNA

**Figure 6:**
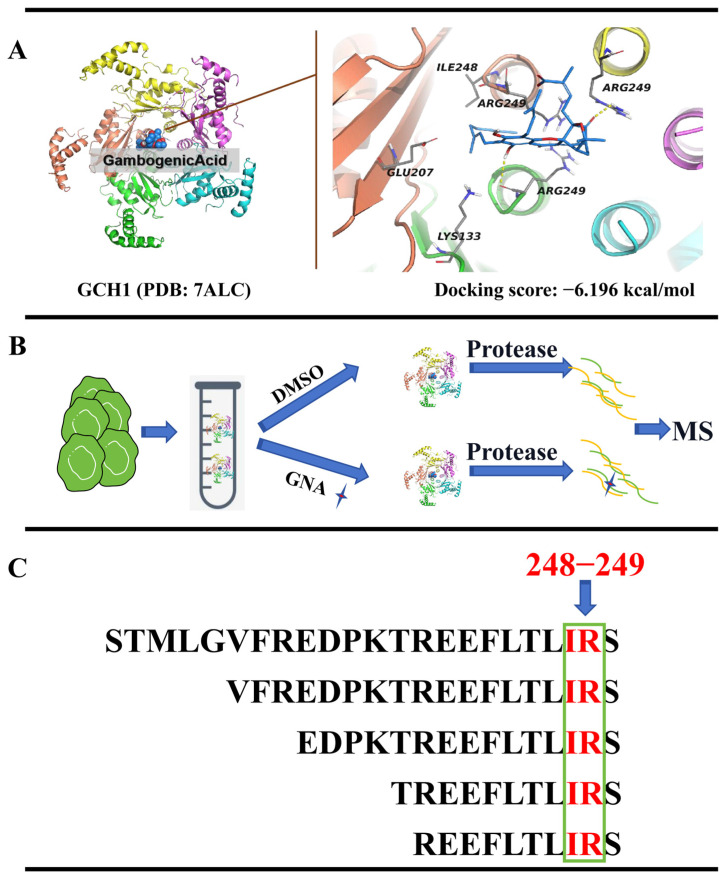
GCH1 is the downstream target of GNA. (**A**) GNA is linked to GCH1 active site via hydrogen bonds to form a complex. (**B**,**C**) Flow chart depicting LiP-SMap assay. Freshly prepared whole-cell lysates were treated with or without GNA followed by proteinase K (PK) digestion and MS analysis. GCH1 binding prevents PK digestion, leading to differential MS peptide profiling.

**Figure 7:**
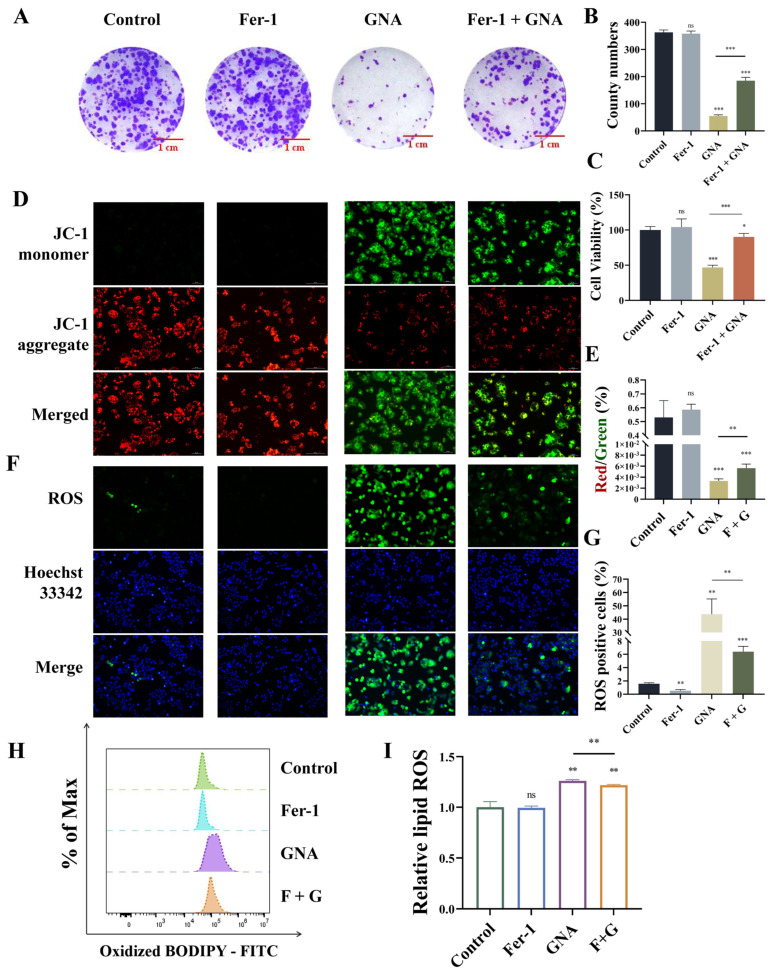
GNA treatment inhibits malignant progression of NSCLC by promoting ferroptosis. (**A**,**B**) Colony formation assay results for clone numbers in HCC1833 cells. (**C**) CCK-8 results for HCC1833 proliferation. (**D**,**E**) Immunofluorescence results for mitochondrial membrane potential (20×). (**F**,**G**) Immunofluorescence results for ROS deposition (20×). (**H**,**I**) BODIPY 581/591 results for lipid peroxidation. Data are expressed as mean ± SD. ns: no significance, * *p* < 0.05, ** *p* < 0.01, *** *p* < 0.001 vs. control.

To determine whether GCH1 was a target of GNA, GNA and GCH1 were docked utilizing the DockThor molecular docking tool. The outcomes showcased that GNA directly bound to the active site of GCH1 via multiple hydrogen bonds to form complex with a binding energy −6.196 kcal/mol (Figure 6A). GNA interacted with LYS133, GLU207, ILE248, and ARG249 of GCH1. To identify potential targets, LiP-SMap was utilized to identify cellular proteins binding to GNA directly. C3H10T1/2-derived HCC1833 lysates were incubated with GNA or dimethyl sulfoxide, followed by limited proteolysis with proteinase K and trypsin, and LC-MS/MS (Figure 7B). The results showed that ILE248 and ARG249 of GCH1 interacted with GNA (Figure 6B,C), suggesting that GCH1 was a downstream target of GNA.

### 2.6. GNA Suppressed Malignant Progression of NSCLC by Enhancing Ferroptosis

Next, the present study aimed to reveal whether GNA suppressed malignant progression of NSCLC by enhancing ferroptosis. Treatment with 1 μM ferrostatin-1 (Fer-1) significantly restored proliferation ability after GNA treatment, as determine with the colony formation assay (Figure 7A,B). CCK-8 assay data also showcased that Fer-1 treatment significantly restored proliferation ability after GNA treatment (Figure 7C). The immunofluorescence results revealed that Fer-1 treatment significantly restored inhibition of mitochondrial membrane potential after GNA treatment (Figure 7D,E). It has been suggested that GCH1/DHFR is an exclusive mechanism for protection against ferroptosis independent of the GPX4/glutathione system, which may be associated with the antioxidant defense system [21]. Therefore, the total ROS and cell lipid peroxidation levels were assessed. The immunofluorescence data illustrated that Fer-1 treatment significantly reversed promotion effects regarding GNA during ROS deposition (Figure 7F,G). The flow cytometry results suggested that Fer-1 treatment significantly reversed GNA-induced oxidation of liposomes (Figure 7H,I), suggesting that GNA treatment suppressed malignant progression of NSCLC by promoting ferroptosis.

### 2.7. GCH1 Overexpression Reversed the Inhibitory Effects of GNA Against Malignant Progression of NSCLC by Regulating Ferroptosis

**Figure 8:**
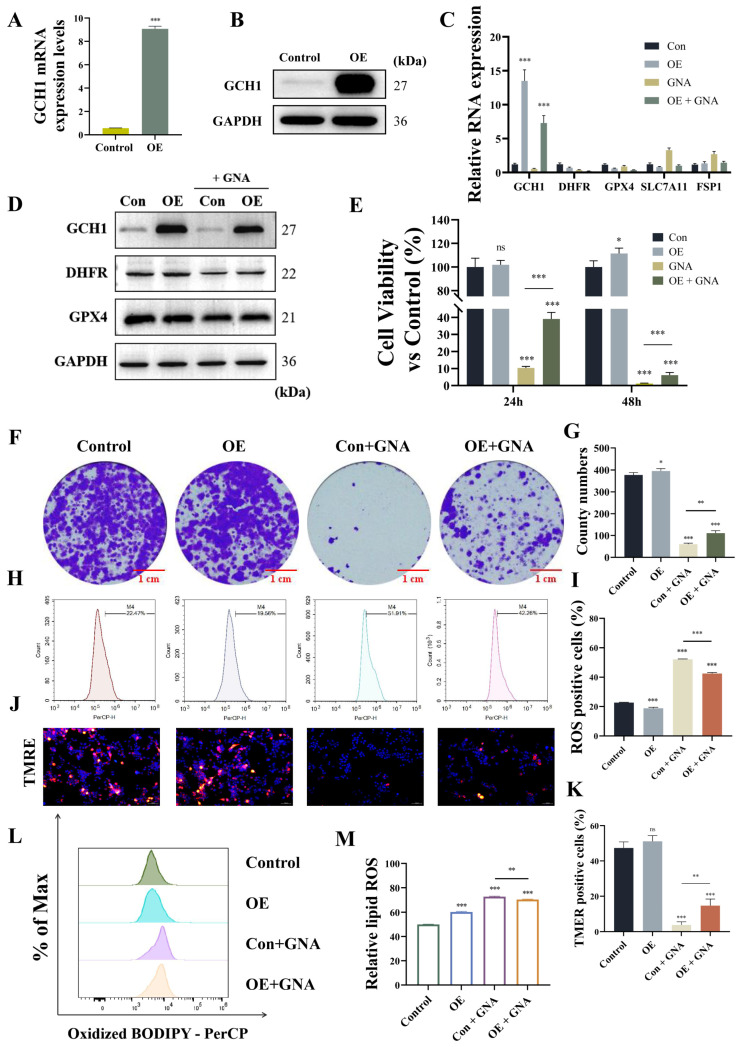
GCH1 overexpression reverses inhibitory effect of GNA on malignant progression in NSCLC by regulation ferroptosis. (**A**) RT-qPCR results for GCH1 expression in HCC1833 cells after transfection with GCH1 overexpression vector. (**B**) Western blot results for GCH1 expression. (**C**) RT-qPCR results for ferroptosis-related gene expression. (**D**) Western blot results for ferroptosis-related protein expression. (**E**) CCK-8 results for proliferation ability of HCC1833 cells. (**F**,**G**) Colony formation assay results for clone numbers in HCC1833 cells. (**H**,**I**) Flow detection of oxidative stress in HCC1833 cells after different treatments. (**J**,**K**) Immunofluorescence results for tetramethylrhodamine ethyl ester perchlorate staining show mitochondrial membrane potential (20×). (**L**,**M**) BODIPY 665/676 results for lipid peroxidation. Data are expressed as mean ± SD. ns: no significance, * *p* < 0.05, ** *p* < 0.01, *** *p* < 0.001 vs. control.

Next, we investigated whether GNA suppressed malignant progression of NSCLC by targeting GCH1. Transfection of HCC1833 cells with the GCH1 OE vector significantly increased GCH1 expression at mRNA and protein levels (Figure 8A,B). GCH1 overexpression restored mRNA and protein levels regarding GCH1 and DHFR after GNA treatment but did not significantly affect other expressions of ferroptosis-related genes (Figure 8C,D). The results of the CCK-8 and colony formation assays verified that GCH1 overexpression restored proliferation ability post GNA treatment (Figure 8E–G). Flow cytometry demonstrated that GCH1 overexpression significantly reversed GNA-induced oxidation of liposomes (Figure 8H,I). The results of the mitochondrial membrane potential assay with TMRE staining revealed that GCH1 overexpression significantly restored inhibition of mitochondrial membrane potential after GNA treatment (Figure 8J,K), suggesting that GCH1 overexpression reversed the inhibitory effects of GNA against malignant progression of NSCLC by regulating ferroptosis.

### 2.8. GCH1 Overexpression Reversed GNA Inhibitory Effects

**Figure 9:**
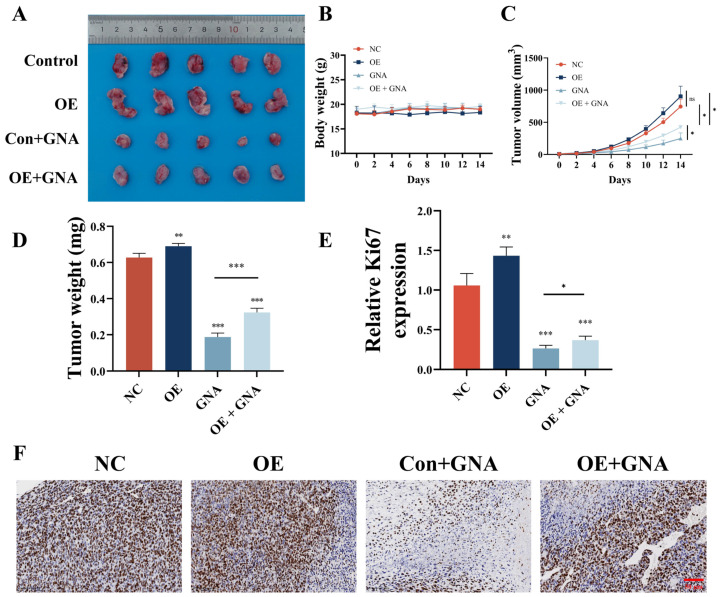
GCH1 overexpression reverses inhibitory effect of GNA on tumor growth in NSCLC. (**A**) Representative HCC1833 tumor formation images in nude mouse xenografts. (**B**) Tumor weight was determined 14 days after injection. (**C**) Mouse body weight was determined every two days for 14 days. (**D**) Tumor volumes were recorded every two days. Data are expressed as means ± SD. ** *p* < 0.01, *** *p* < 0.001 vs. NC. (**E**,**F**) Immunohistochemical analysis showing percentage of Ki67-positive cells. Data are expressed as mean ± SD. ns: no significance, * *p* < 0.05, *** *p* < 0.001 vs. control.

GCH1 overexpression reversed GNA inhibitory effects against tumor growth (Figure 9A). GCH1 overexpression or in combination with GNA treatment did not influence the body weight of mice (Figure 9B). However, GCH1 overexpression reversed the GNA inhibitory effects upon tumor weight (Figure 9C) and tumor volume (Figure 9D). Immunohistochemical analysis of Ki67 expression showed that GCH1 overexpression reversed the GNA inhibitory effects against Ki67 expression in tumor tissues (Figure 9E,F).

## 3. Discussion

Studies have confirmed that the natural compound GNA can induce apoptosis and ferroptosis in melanoma and osteosarcoma [4,14]. In present research, GNA inhibited malignant progression regarding NSCLC by suppressing cell proliferation and migration, tumor growth, and pulmonary metastasis. The half-maximal inhibitory concentration of GNA was lower in pulmonary epithelial BEAS-2B cells than in other LC cells, suggesting that tumor cells were more sensitive to GNA than normal cells and that GNA was suitable as an anticancer drug.

Cell cycle analysis confirmed that GNA significantly arrested A549 and HCC1833 cells in the G0/G1 phase. Apoptosis assay data showcased that GNA enhanced apoptosis regarding both A549 and HCC1833 cells, suggesting that GNA directly killed tumor cells. Previous studies also confirmed that GNA suppressed cell viability and induced cell cycle arrest at the G0/G1 phase in both choroidal melanoma and LC [13,22]. Moreover, GNA was known to enhance gemcitabine-induced apoptosis and improve tumor cell resistance by enhancing the expression of apoptosis-related proteins [23]. Therefore, present investigation outputs were clinically significant.

Proteomic detection data showed that GNA treatment resulted in a metabolic disorder, and GCH1 is a rate-limiting enzyme [24]. GCH1 deficiency resulted in decreased BH4 levels [25,26]. Prior investigations confirmed that GCH1 expression was positively correlated to the infiltration of regulatory T cells [9,27]. In current research, GNA treatment suppressed expression of GCH1. GNA and GCH1 were docked utilizing the DockThor online molecular docking tool. LiP-SMap showed that GNA interacted with ILE248 and ARG249 of GCH1, suggesting that GNA suppressed malignant progression of NSCLC by targeting GCH1.

Several studies have confirmed that GCH1 suppression and erastin co-treatment in vivo synergistically suppressed tumor growth [28]. In addition, inhibition of GCH1 effectively increased lipid peroxidation and induced ferroptosis [8]. The ferroptosis inhibitor Fer-1 was used to determine whether GNA induced ferroptosis. Data showcased that Fer-1 treatment restored proliferation and migration abilities regarding NSCLC cells post-GNA treatment, suggesting that GNA suppressed malignant progression of NSCLC by inducing ferroptosis. Immunofluorescence and flow cytometry showed that GNA induced ROS deposition and decreased mitochondrial membrane potential, resulting in lipid peroxidation and oxidative stress. Fer-1 treatment also induced mitochondrial oxidative stress and attenuated ferroptosis [29].

Further study found that GCH1 overexpression had a similar effect to that of Fer-1. GCH1 overexpression restored proliferation and migration abilities regarding NSCLC cells after GNA treatment as well as mitochondrial membrane potential and inhibited ROS deposition. GCH1 and BH4/BH2 led to lipid remodeling, thereby inhibiting ferroptosis. GCH1 expression levels in cancer cells stratified susceptibility to ferroptosis [21,30]. The in vivo experimental data showed that GCH1 overexpression reversed GNA inhibitory effects against tumor growth, suggesting that GNA suppressed malignant progression regarding NSCLC through targeting GCH1 and inducing ferroptosis.

In summary, the outcomes of the current study unraveled that GNA suppressed NSCLC malignant progression via inducing ferroptosis and targeting GCH1. To maximize the anti-tumor effect, GNA is mainly used in combination with chemotherapy drugs. Studies have found that the combination of glycolic acid and daunorubicin could reverse the resistance of lymphoma cells to daunorubicin by down-regulating P-glycoprotein levels [31]). In addition, GNA could increase glioblastoma multiforme cell sensitivities to temozolomide by specifically inhibiting BMI1 [32]. Therefore, GNA is a potential antitumor agent for NSCLC treatment.

## 4. Materials and Methods

### 4.1. Ethics Statement

The research was approved by Institutional Animal Care and Use Committee in Shanghai University of Medicine and Health Sciences (no. 2024-GZR-16-340406198707142817) following “Guide for the Care and Use of Laboratory Animals”.

### 4.2. Experimental Mice

BALB/c nude mice (4~6 weeks old; 15~20 g weight) were obtained in Shanghai Laboratory Animal Research Center (Shanghai, China) and housed in animal care facility with constant temperature with free access to food and water.

### 4.3. Cell Culture

A549, HCC1833, H1650, and pulmonary epithelial BEAS-2B cells were donated by the Chinese Academy of Sciences (Shanghai, China) and cultivated in DMEM (Shanghai Basal Media Technologies Co., Ltd., Shanghai, China) supplemented with penicillin-streptomycin and 10% FBS (Gibco^®^; Invitrogen Corporation, Carlsbad, CA, USA) under atmosphere of 5% CO_2_/95% air and transfected with a lentivirus construct overexpressing GCH1 (OE) or a negative control (NC) vector. RNA was extracted from passaged cells to validate successful transfection.

### 4.4. Quantitative Real-Time Polymerase Chain Reaction (qRT-PCR)

Total RNA was isolated by an RNAfast200 kit (Shanghai Fastagen Biotechnology Co., Ltd., Shanghai, China), quantified with NanoDrop spectrophotometer (Thermo Fisher Scientific, Waltham, MA, USA), and reversely transcribed into complementary DNA using a commercial kit (Takara Bio, Inc., Kusatsu, Japan) with gene-specific primers (Table S1; Sangon Biotech Co., Ltd., Shanghai, China). Relative gene expression levels were detected by 2^-ΔΔCt^ approach. All qRT-PCR analyses were conducted using StepOnePlus RT-PCR System (Thermo Fisher Scientific) and SYBR Green PCR Master Mix (Yeasen, Shanghai, China). Thermal cycle parameters included 40 cycles of denaturation annealing at 60 °C.

### 4.5. Molecular Docking Analysis

The target genes were identified based on the proteomics results. The crystal structures of relevant proteins and small-molecule compounds were elucidated from Protein Data Bank (https://www.wwpdb.org/, accessed on 3 February 2025) and PubChem database (https://pubchem.ncbi.nlm.nih.gov/, accessed on 3 February 2025). Molecular docking was performed using the online tool DockThor (https://dockthor.lncc.br/v2/, accessed on 3 February 2025).

### 4.6. Liquid Chromatography-Mass Spectrometry (LiP-SMap)

HCC1833 cells were seeded in 10 cm dishes with or without GNA (1 μM). After cleaning with PBS, cells were lysed via radioimmunoprecipitation assay buffer including 1 mM phenylmethylsulfonyl fluoride. Protein concentration was detected utilizing bicinchoninic acid assay (Pierce™ BCA Protein Assay Kit; Thermo Fisher Scientific). Lysates were maintained at 80 °C. To extract 250 μg of total protein, we reduced samples in 10 mM dithiothreitol and alkylated for 1 h at 25 °C. Next, 1.5 mL pre-cooled 100% acetone was added to clean precipitate and samples were then centrifuged three times at 14× *kg* for 15 min, air-dried for 2~3 min, and diluted. Peptides were desalted with SPE C [18] cartridges (Thermo Fisher Scientific) and then lyophilized in a vacuum before analysis by liquid chromatography-tandem mass spectrometry (LC-MS/MS).

### 4.7. Ethynyl Deoxyuridine (EdU) Assay

Cell proliferation was detected with EdU Cell Proliferation Assay kit (UE, Shanghai, China). HCC1833 and A549 cells were seeded in the wells of 24-well plates and cultivated to 60–70% confluency. After adding EdU labeling reagent to individual wells, cells were incubated for 2 h, then fixed in 4% paraformaldehyde, permeabilized with 0.1% Triton X-100 (MCE, New Jersey, USA), and stained with Hoechst 33342 (UE, Shanghai, China).

### 4.8. Plate Colony Formation Assay

Suspended A549 and HCC1833 cells (1 × 10^3^) following different treatments were added to the wells of 6-well plates and cultured for 2 weeks. Cells were imaged with a fluorescence microscope throughout the experiment. Finally, cells were washed twice with PBS, stained with Giemsa dye at 500 µL/well for 10~20 min, cleaned three times with PBS, and photographed by digital camera (Leica Camera Inc., Teaneck, NJ, USA).

### 4.9. Cell Counting Kit-8 (CCK-8) Assay

Cells were incubated in 10% CCK-8 solution diluted in normal culture medium at 37 °C. Proliferation rates were obtained at 1, 2, and 3 days after treatment. Absorbance of every well was measured with microplate reader at 450 nm.

### 4.10. Apoptosis Assay

Apoptosis was detected with fluorescein isothiocyanate-annexin V assay using commercial kit (BD Biosciences, Franklin Lakes, NJ, USA). Following removal of non-adherent cells, adherent cells were washed twice, and trypsinized for 3 min. Next, 1 × 10^5^ cells were centrifuged at 1000 rpm, supernatant was discarded, and the resulting pellet was resuspended in 100 μL 1× binding buffer in Eppendorf tubes. Finally, 5 μL each of propidium iodide and annexin V-allophycocyanin were put to each tube. Post mixing, the cells were incubated with room temperature, followed by the addition of 500 μL 1× binding buffer to stop the reactions.

### 4.11. Cell Cycle Assay

Cell cycle analysis was made by Cell Cycle and Apoptosis Analysis kit (Beyotime Biotechnology, Shanghai, China). In brief, cells were cleaned, resuspended in 500 μL of staining buffer, 10 μL of RNase A (50×), and 25 μL of propidium iodide staining solution in Eppendorf tubes. Cell cycle stages were identified utilizing a low-loading-speed flow cytometer (Agilent Technologies, Inc., Santa Clara, CA, USA).

### 4.12. Wound Healing (WH) Assay

Control and transduced cells were added to the wells of 6-well plates and cultivated in DMEM to confluency of 90–95%. Then, the cell monolayer was scratched with 1 mL pipette tip and detached cells were erased. Wound area was imaged on days 0, 1, and 2 post-seeding and analyzed by ImageJ1.8.0. Cell scratch healing rate was computed.

### 4.13. Transwell Migration Assay

In brief, 200 μL serum-free medium containing 5 × 10^4^ treated cells were added to upper Transwell chamber (BD Biosciences) and 500 μL complete medium were put to lower chamber. The cells were incubated for one day, imaged under inverted light microscope (Primovert; Carl Zeiss AG, Jena, Germany), and quantified by ImageJ1.8.0.

### 4.14. Reactive Oxygen Species (ROS) Measurement

#### 4.14.1. Total ROS Measurement

Under the ROS action, 2′7′-dichlorofluorescin (DCFH) was oxidized to 2′7′-dichlorofluorescein (DCF), as confirmed by emission of green and red fluorescence, respectively (UElany Inc., Suzhou, China). Cells were treated as indicated and then incubated with 1 μM DCFH/DHE, while protected from light. The dye was then removed and replaced with fresh PBS. Cellular ROS levels were quantified by fluorescence microscopy and flow cytometry.

#### 4.14.2. Lipid ROS Measurement

The appropriate BODIPY 581/591 or BODIPY 665/676 fluorescent dye (Thermo Fisher Scientific) was selected based on the tested cell type. Cells were treated as indicated and then incubated with 20 μM BODIPY 581/591 or BODIPY 665/676 staining solution. Lipid peroxidation levels indicating ferroptosis were detected by flow cytometry.

### 4.15. JC-1 Measurement

When mitochondrial membrane potential is high, JC-1 (UE, Shanghai, China) will aggregate into polymers that emit red fluorescence. If mitochondrial membrane potential is low, JC-1 occurs as monomer generating green fluorescence. HCC1833/OE cells were cultivated in the wells of 24-well plates containing 10% fetal bovine serum. After one day of treatment with GNA (1 μM), cells were cleaned by PBS and incubated with JC-1. Fluorescence was detected by fluorescence microscope with excitation wavelength of 490 nm for JC-1 monomers and 525 nm for polymers. Changes to mitochondrial membrane potential were assessed by comparing the fluorescence intensity and color under different conditions.

### 4.16. TMRE Measurement

Mitochondria in living HCC1833/OE cells were labeled with fluorescent probe tetramethylrhodamine ethyl ester perchlorate (TMRE; 100 nM; MedChemExpress, Monmouth Junction, NJ, USA) in working solution protected from light. Red fluorescence of TMRE was detected with a fluorescence microscope.

### 4.17. In Vivo Experiments

Animal experiments were performed following (8). NSCLC nude mouse models were generated by flank injection of HCC1833 cells with NC or GCH1 overexpression (OE) vectors.

To obtain tumor metastasis data, nude mice were injected via tail with luminescence-labeled HCC1833 cells re-suspended in sterile PBS. Lung metastasis was confirmed after 12 days utilizing in vivo bioluminescence imaging. Metastatic foci in lung tissues were quantified by staining with hematoxylin and eosin.

### 4.18. Western Blot (WB) Analysis

Total cell protein contents were harvested by cold radioimmunoprecipitation assay buffer, quantified by BCA protein assay kit, separated by electrophoresis, and electroblotted onto polyvinylidene fluoride membranes, which we incubated with antibodies against GCH1, DHFR, GPX4, and GAPDH (Abcam Limited, Cambridge, UK).

### 4.19. Statistical Analysis

All data are reported by mean ± standard deviation. Differences between groups were assessed with Prism (GraphPad9.5.1 Software, Inc., La Jolla, CA, USA). *p*-value ≤ 0.05 was regarded as statistically significant.

## 5. Conclusions

Overall, the present study found that GNA treatment suppressed malignant progression of NSCLC via induced GCH1-mediated ferroptosis. GNA-induced ferroptosis is independent of the classical GPX4 pathway and may be related to lipid peroxidation caused by GCH1 inhibition. As a limitation to this study, how GNA targets GCH1 and inhibits GCH1 expression remains unclear. We are therefore planning an additional study to clarify the specific mechanism of GNA to regulate the progression of NSCLC.

## Data Availability

The data discussed in present investigation are included within the paper and Supplementary Files.

## Supplementary Materials

The following supporting information can be downloaded at: https://www.mdpi.com/article/10.3390/ph18030374/s1, Table S1: Primer sequences used in this study.

## Abbreviations

BH4, Tetrahydrobiopterin; DHFR, Dihydrofolate reductase; EdU, Ethynyl deoxyuridine; GCH1, GTP cyclohydrolase 1; GNA, Gambogenic acid; NSCLC, Non-small cell lung cancer; WH, Wound healing.
