# Supplementary material for: Gambogenic Acid Suppresses Malignant Progression of Non-Small Cell Lung Cancer via GCH1-Mediated Ferroptosis
Source: Pharmaceuticals (Basel). 2025 Mar 6;18(3):374. doi: 10.3390/ph18030374 (PMC11944504; doi:10.3390/ph18030374)
Supplement: Supplementary file 1 [file pharmaceuticals-18-00374-s001.zip › pharmaceuticals-3481249-supplementary.pdf]

Table S1. Primer sequences used in this study

**Primers used for qPCR:**	
ACAT2 Forward	CATGCTGCTGCTCATCTTCT
ACAT2 Reverse	ACTGCGGAGACCAGGAACA
PTGS2 Forward	GGGAGTCTGGAACATTGTGAA
PTGS2 Reverse	GTGCACATTGTAAGTAGGTGGACT
GCH1 Forward	ACAAGCAAGTCCTTGGTCTCA
GCH1 Reverse	GTGAGGCGCTCTTGAACTTG
ALAS1 Forward	TCAACCCTCTTCACCCTGGCTAAG
ALAS1 Reverse	TACTTTGGCACTCGGCTGTTTCG
UAP1L1 Forward	GGAGCGGAAAGACAAAGTTGC
UAP1L1 Reverse	CACAGAAGCCGATGAAGACAGG
IDH1 Forward	TGCAAAAATATCCCCCGGCT
IDH1 Reverse	TACATCCCCATGGCAACACC
HMGCS1 Forward	TGTACACATCTTCAGTATATGGTTCCC
HMGCS1 Reverse	AAGAAAACACTCCAATTCTCTTCCCT
B4GalT5 Forward	CCGCTCGAGTGGCTGCAGCATGCGCG
B4GalT5 Reverse	CGCGGATCCTCAGTACTCGTTCACCTGAG
CCDC170 Forward	TCAAAGATGTGACTACTGGGCAAGA
CCDC170 Reverse	GAAGAGCAAGGCTGGTCACGT
KRT80 Forward	GCTGCTCTTGCCATAATCAA
KRT80 Reverse	AATGCTCCTGCCCAATCTC
APOB Forward	GCAACACCTCTTCCTGCCTTTCT
APOB Reverse	GGCCCATCTTCTTAGTACCTTCACC
GBP5 Forward	CCATGTGCCTCATCGAGAACT
GBP5 Reverse	ACAGGTTGCGTAATGGCAGAC
STAMBPL1 Forward	GAGGATGGCGTCTGTGTATTTG
STAMBPL1 Reverse	GCTGGTAATCTCGATGGTTAGG
CDKN2D Forward	ATGCTGCTGGAGGAGGTTCGC
CDKN2D Reverse	CGTCTTGCCGAAGCGGTTGAG
ATP6V1B2 Forward	GGGCAAGTTCTGGAAGTTAGT
ATP6V1B2 Reverse	TACTGCCTTGGAACCACTAAC
B4GalT5 Forward	TGGAACAGAGTACAGAATGCAG
B4GalT5 Reverse	CCTTGCCGTTCTTTTGACTTC

GPX4 Forward	TGGGAAATGCCATCAAGTGG
GPX4 Reverse	GGTCCTTCTCTATCACCAGGGG
SLC7A11 Forward	TCTCCAAAGAGGTACCTGC
SLC7A11 Reverse	AGACTCCCCTCAGTAAAGTGAC
FSP1 Forward	GCCAACATCGTCAACTCT
FSP1 Reverse	CCACATAGAAGCCACTGAT
DHFR Forward	TCCAGAGAATGACCACAAC
DHFR Reverse	GCCACCAACTATCCAGAC
β-actin Forward	CCTGTACGCCAACACAGTGC
β-actin Reverse	ATACTCCTGCTTGCTGATCC
